# Enhanced Stress-Resilience Training (ESRT) for graduate-entry medical students: a randomised-controlled, mixed-method investigation

**DOI:** 10.1186/s12909-025-07768-6

**Published:** 2025-09-02

**Authors:** Luke Sanders, Georgie Budd, Lebares Carter, Umakant Dave, Andrew H. Kemp

**Affiliations:** 1 School of Psychology, Swansea University, Swansea, UK; 2 Medical School, Swansea University, Swansea, UK; 3 UCSF School of Medicine, University of California San Francisco, San Francisco, USA; 4 Morriston Hospital, Swansea, UK

**Keywords:** *Enhanced Stress-Resilience training*, *Medical students*, *Psychological flexibility*, *Resilience*, *Stress reactivity*

## Abstract

**Background:**

Medical students face demanding academic requirements, fierce competition, self-doubt and financial concerns contributing to high rates of depression, anxiety, stress, sleep problems and burnout, highlighting a need for effective interventions. We explored an intervention called Enhanced Stress-Resilience Training (ESRT), a modified form of mindfulness training adapted for clinicians, that was applied to medical students for the first time.

**Methods:**

Graduate-entry medical students (*N* = 118) were randomised to ESRT or an active control condition as part of a registered trial (ISRCTN16324994). A 3 (Time: pre-, post-, and six-month follow-up) × 2 (Group: ESRT, control) mixed design was used to assess changes in psychological flexibility, resilience, and stress reactivity. Due to attrition (T3 *n* = 47), additional sensitivity analyses, including intention-to-treat and subgroup analyses based on baseline resilience, were conducted. Qualitative data from exit evaluations (*n* = 25), interviews (*n* = 12), and focus groups (*n* = 11) underwent reflexive thematic analysis to explore student experience and contextualise quantitative findings.

**Results:**

ESRT users demonstrated increased psychological flexibility scores at post-intervention (*p* = .001, *d* = 0.62) and six-months follow-up (*p* = < 0.001, *d* = 0.96), and these findings were confirmed in intention-to-treat analysis. Exploratory analyses revealed that those with low baseline resilience who underwent ESRT displayed increased resilience (*p* = < 0.001, *d* = 1.86) and decreased stress reactivity (*p* = < 0.001, *d* = 1.58) at the six-month follow-up. Qualitative findings highlighted high acceptability, perceived value, and a strong desire for curricular integration, while also revealing barriers to engagement, particularly time constraints.

**Conclusions:**

This study offers new evidence that ESRT may enhance psychological flexibility and resilience, and decrease stress reactivity, especially among more vulnerable students. Sustained effects and positive qualitative feedback suggest that curricular integration could improve feasibility and reach.

**Supplementary Information:**

The online version contains supplementary material available at 10.1186/s12909-025-07768-6.

## Introduction

Medical students battle heavy workloads, fierce competition, and a wide range of psychological and behavioural symptoms (PBS) including sleep problems (42.0%), stress (41.7%), burnout (35.8%), depression (32.5%), anxiety (32.5%), internet addiction (26.0%), and substance use (25.2%) [1]. This umbrella review and meta-analysis of meta-analyses concluded that a third of medical students self-reported a wide range of PBS, globally. Such findings risk damaging the quality of patient care and patient safety [2, 3] highlighting an urgent need for effective interventions for service providers.

This concern was magnified during the COVID-19 pandemic, which saw rates of depression and anxiety among medical students surpass those of the general population and qualified healthcare workers [4]. The rapid transition to remote learning disrupted clinical rotations [5, 6] and left students feeling underprepared for future clinical practice [7, 8]. Yet qualitative reports from medical students also acknowledged that the pandemic was characterised by decreases in mental health stigma and increases in awareness-raising for mental health support [9]. Indeed, medical students have expressed a demand for post-COVID curricular innovations to support their clinical training [10], while researchers have argued that the adaptations provoked by the COVID-19 pandemic could facilitate ongoing positive transformations to medical education [5, 8].

Mindfulness-Based Stress Reduction (MBSR) [11, 12] and derived Mindfulness-Based Interventions (MBIs) [11, 13] have become popular strategies for driving these changes. Designed as weekly group-based systematic training programmes for developing a range of physical and mental mindfulness practices, the goal of MBSRs and MBIs is to teach users self-regulation skills that cultivate acceptance and non-reactivity [14, 15]. By offering users skills in meditation, body scan exercises, and mindful breathing, such courses have been shown to reduce medical student PBS and enhance wellbeing and self-compassion [16–18]. However, implementation has been hindered by limited financial support, mental health stigma and concerns regarding the feasibility of such programmes for a student population who already struggle with time constraints and demanding curricula [19–21].

Enhanced Stress-Resilience Training (ESRT) is a tailored MBI developed using human-centred design and implementation science techniques to increase acceptability and accessibility for time-compressed, high-stakes occupations [22, 23]. Such tailoring aligns with a broader trend in resilience science and among mindfulness researchers to expand the reach of MBIs to enhance healthcare worker resilience [24–26]. ESRT draws inspiration from the seminal ‘broaden and build’ model [27–29], which suggests that positive emotions can expand an individual’s thought-action repertoire by developing psychological resources such as mindfulness, flexibility, and resilience. In line with this framework, the goal of ESRT is to cultivate three key cognitive resources: meta-cognition, emotion regulation, and interoception.

By condensing the traditional MBI model of 8-weekly 2.5 h sessions into 5 weekly one hour sessions, with content and delivery tailored to medical personnel, ESRT has been shown to improve executive functioning in US-based surgical trainees and to decrease burnout and physiological distress as measured through pro-inflammatory RNA levels [30]. Luton and colleagues [31] reported that the intervention generalised well to UK surgical trainees, observing reductions in stress and increased mindfulness. More recently, the same authors reported findings from an 18-month follow up of their original investigation [32]. In that study, the authors observed a 13-fold better career progression among those who participated in ESRT, as well as an increase in mindfulness and reductions in burnout and stress longitudinally.

While ESRT has proved beneficial and exhibited cross-cultural generalisability between US [30, 33] and UK surgical trainees [31, 32], it remains unclear whether the benefits will generalise to graduate-entry medical students. The unique challenge of transitioning from undergraduate studies to graduate medicine calls for radical knowledge acquisition [34–36] and students must embark on clinical rotations where they face uncertainty, fears of appearing incompetent, and the realities of human suffering [35, 36]. Since ESRT has been iteratively adapted to navigate the unique culture and infrastructure of graduate-level surgical training [22], we suspected the programme would adapt well to graduate-entry medical students. In unpublished pilot work, we evaluated ESRT in 60 US medical students at schools participating in the national Learning Communities Institute and received positive subjective feedback regarding acceptability and relevance.

We therefore conducted a randomised control trial investigating the effectiveness of ESRT for graduate-entry medical students at Swansea University in Wales, UK. We measured effectiveness via a questionnaire comprised of outcome measures focused on key psychological resources relating to resilience and adaptability. These included psychological flexibility, resilience, and stress reactivity. Psychological flexibility refers to the capacity to be present and engage with and manage feelings, thoughts, and emotions, and is considered a key mechanism through which MBIs improve wellbeing [37], and has been shown to mediate the effect of self-esteem on PBS among medical students [38]. Resilience refers to the ability to adapt to and recover from stressors, which partially mediates the relationship between mindfulness and burnout [39, 40] and depression [41], while mediating the relationship between MBIs and PBS reductions [42]. Finally, stress reactivity refers to dispositional change in physiological and psychological stress responses, positively correlating with PBS such as depression and rumination [43], predicting self-critical perfectionism [44], and moderating the relationship between blood pressure and reported task demand and control among medical students [45]. Non-reactivity facilitated by mindfulness practice may underpin reductions in perceived stress [15] including among healthcare providers [46, 47].

As such, we hypothesised that in comparison to controls, ESRT users will exhibit significantly higher levels of psychological flexibility and resilience, and lower levels of stress reactivity by post-intervention. To contextualise our results, student testimonies regarding their experience of ESRT were gathered through exit evaluations, one-on-one interviews, and end-of-study focus groups using a mixed method approach to integrate quantitative and qualitative findings [48–50]. Findings illustrate the effectiveness of ESRT for medical students and highlight obstacles to future implementation efforts as well as some considerations and suggestions on how these obstacles might be overcome.

## Method

### Design

**Fig. 1:**
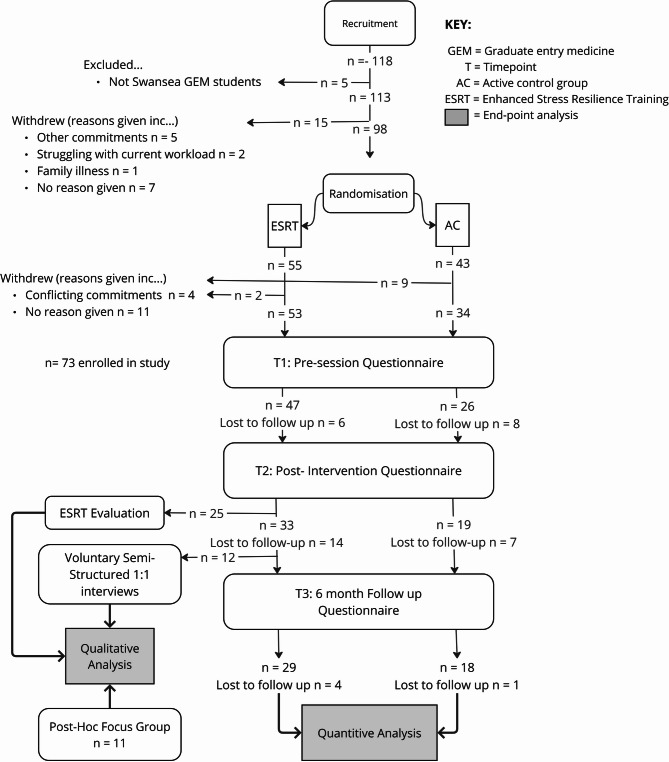
Participant Flow

This study was originally designed as a fully powered randomised controlled trial to evaluate the effectiveness of ESRT in graduate-entry medical students. A priori power analysis conducted using G*Power determined a target of 167 participants (based on a moderate effect size (f = 0.25), α = 0.05, power = 0.80, and an anticipated attrition rate of < 30%. However, due to recruitment challenges and disruptions caused by the COVID-19 pandemic, only 118 participants were enrolled after which 98 participants were successfully randomised to group. Attrition further reduced the final sample at follow-up (T3) to 47 participants (Fig. 1). This issue was addressed through a combination of sensitivity analyses, inspection of Bayes factors and integration of quantitative and qualitative data.

A mixed-method sequential explanatory design [51, 52] was adopted to extend quantitative findings, aligning with recent recommendations for best practice in health research informing public policy [48–50]. Quantitative data were collected according to a 3 Time (Time: pre-intervention [T1], post-intervention [T2], 6-month follow-up [T3]) x 2 Group (Group: ESRT vs. active control) mixed factorial design. Analyses focused on psychological flexibility, resilience, and stress reactivity. Qualitative data were collected through online exit evaluations, one-on-one interviews, and end-of-study focus groups. The inclusion of focus groups was introduced post hoc to elicit shared reflections and better understand contextual barriers to implementation. Reflexive thematic analysis was used to explore key themes relating to student experience, perceived impact, and feasibility, enabling richer interpretation of the intervention’s effects and informing future implementation efforts.

### Participants

Graduate-entry medical students from Swansea University were recruited (*N* = 98) voluntarily from all four academic years of the curriculum. Three recruitment cycles were conducted, spanning academic years 2020/21, 2021/22, and 2022/23, which involved a multi-pronged recruitment strategy that included advertisements via flyers, social media posts, emails to student cohorts, and presentations delivered to students directly by research assistants. Participants were randomly allocated via random number sequencing to either ESRT (*n* = 55, 56%) or active control (*n* = 43, 44%) groups. Following data collection, the active control group were offered ESRT. Active control group participants were informed that they would be offered ESRT at the end of the study.

### Intervention

Three intervention periods were held between 6/10/2021–10/11/2021, 09/02/2022–16/03/2022, and 16/11/2022–14/12/2022. Both groups performed weekly 60-minute sessions of either ESRT or group discussions on current health-related news over a five-week period. Prior to recruitment, the decision was made to deliver all sessions remotely, owing to restrictions relating to the COVID-19 pandemic. ESRT sessions were held by a qualified ESRT practitioner (UD) to guide participants through the ESRT curriculum. Table 1 provides a week-by-week summary of the ESRT intervention.

**Table 1:** Summary of enhanced Stress-Resilience training (ESRT) content

Class No. and Topic	Class Objectives	Class Activities	Didactic Content
Class 1: Resilience	• Define resilience. • Awareness of own resilience. • Recognise the link between thought patterns and resilient outlook/behaviours.	• Breath/body awareness (~ 15 min) • Walking meditation (20 min) • Body scan (20 min)	• Science of resilience; history of resilience training. • Closing discussion: the resilience toolbox.
Class 2: Managing thoughts	• Recognise link between body sensations and emotions. • Recognise link between emotions and thoughts. • Understand that thoughts may feel like facts, but are first and foremost ‘sensations’ of the mind.	• Standing (Shadow) Yoga (30 min) • Lying or Sitting practice (~ 15 min) • MBCT/Hallway exercise (~ 5 min)	• Science of mind-body connection. • Closing discussion: formal vs. informal practice.
Class 3: Thought -Emotion connection	• Gain insight into the role of physical sensations as windows into our thoughts/emotions. • Familiarity with the brain-body connection. • Define meta-cognition, the conscious awareness of one’s cognitive habits • Recognise that meta-cognitive awareness can be used to monitor for bias; interrupt negative cognitive habits; or more deeply rejuvenate.	• Five Min breathing space (10 min) • Qi Gong (20 min) • Sitting or Lying Meditation (20 min)	• Science of cognitive reframing. • Closing discussion: Where, when, and how do we use these techniques?
Class 4: Learning to shift perspective	• Recognise the growing awareness of thought-reflexes and the growing ability to respond rather than react to emotional situations. • Learn the functional and habitual characteristics of a resilient mind.	• Body Awareness/Scan (~ 20 min) • Standing Yoga (15 min) • Ice Cube Experiment (15 min)	• Science of resilience and mindfulness - overlap and synergy. • Closing discussion: What circumstances do you have the ability/space to shift?
Class 5: Mindful communication	• Recognise how being present deepens our awareness of self and others. • Appreciate the relationship of this awareness to Emotional Intelligence. • Understand the ‘3 reciprocal domains of healthcare’ and how these influence burnout, dissatisfaction and resolution of these issues. • Recognise how resilience, self-awareness and emotional regulation can transform anger into a fuel for change.	• Qi Gong (20 min) • Communication Dyad (20 min)	• Science of individual influence on systems-level change. • Closing discussion: Body sensations during conversation - what do you notice when you/they are speaking?

Active control group sessions involved reading and discussing five lay-press articles that explored questions related to the stresses and challenges of medical training. Topics such as humility, mastery, disliking patients, and issues surrounding death and dying were addressed. The group engaged in a shared reading of each article, followed by free-form discussions facilitated by GB. In consultation with students and university staff, sessions were scheduled on Wednesdays at 17:30 to minimise disruptions to clinical placement. Both the ESRT and active control groups continued to receive standard institutional pastoral care over the duration of the project.

### Outcomes

Quantitative outcomes including psychological flexibility, resilience, and stress reactivity were measured via an online questionnaire at T1 baseline (prior to intervention), as well as T2 (five weeks) and T3 (six months) post-intervention timepoints. ESRT participants were also asked to complete an online exit evaluation and invited to remote one-on-one interviews to explore their motivation for participating, experience of the intervention, perceived value of the ESRT course, and recommendations for improving the training. Finally, participants were also invited to end-of-study focus groups to collaboratively reflect on their experience and identify barriers to implementation. Students participating in focus groups received £20 Amazon vouchers.

#### Psychological flexibility

Psychological Flexibility was assessed via the Acceptance and Action Questionnaire 2 (AAQ2) [38, 53], which exhibits strong convergent and discriminant validity, while also being robust to social desirability bias. Across six samples, mean Cronbach’s alpha scores were 0.84, indicating strong internal consistency. Test-retest reliability was also solid at both 3-month (α = 0.81) and 12-month (α = 0.79) follow-ups. The AAQ2 consists of 7 items, for which responses are recorded using a Likert scale ranging from 1 (never true) to 7 (always true). Total scores range from 7 to 49, with lower scores indicating greater levels of psychological flexibility.

#### Resilience

Resilience was assessed via the 10 item version of Connor-Davidson Resilience Scale (CD-RISC 10) [54–56]. The scale shows good internal consistency (α = 0.85) and improved construct validity over its original 25-item scale [57]. The scale contains 10 items which are rated on a five-point Likert scale ranging from 0 (not true at all) to 4 (true nearly all of the time). Total scores range from 0 to 40, with higher scores indicating higher levels of resilience.

#### Stress reactivity

Stress Reactivity was assessed via the Perceived Stress Reactivity Scale (PSRS) [44, 45, 58], which exhibits strong item homogeneity, test–retest reliability, and internal consistency (Cronbach’s alpha values primarily ranging from 0.70 to 0.80). The scale consists of 23 incomplete statements which participants complete using one of three suggested answers. For example, item 12: “When something does not go the way I expected…” can be responded with one of the following: “I usually stay calm”, “I often get uneasy”, or “I usually get very agitated”. Answers are coded from 0 to 2 in order of appearance. Reverse scoring is applied for items 2, 5, 8, 10, 11, 13, 15, 17, 18, 19, 20 and 22. Total scores range from 0 to 46, with lower scores indicating lower levels of stress reactivity.

### Apparatus & materials

ESRT and active control sessions, one-on-one interviews, and end-of-study focus groups were all conducted over Zoom. Interview and focus group schedules are available on the Open Science Framework [59]. Questionnaires and exit evaluations were administered via Qualtrics [60]. Qualitative data were analysed using Nvivo (version 1.5.1) [61] – a widely used computer assisted qualitative data analysis software produced by QSR International which significantly reduces the number of manual tasks required in handling qualitative data, providing researchers more time to engage in analysis [62, 63]. Quantitative data were analysed using JASP (version 0.17.2.1) [64] – an open-source statistical analysis software which has been sponsored by the Centre for Open Science and the European Research Council. The software gives researchers the capability of conducting frequentist or Bayesian statistical analyses [65, 66], making it an ideal software for use in our study, which reports on both.

### Randomisation

#### Random sequence generation

A simple randomisation procedure was implemented, using a random number sequence generator in Microsoft Excel, ensuring unbiased group allocation. The sequence was created by a research collaborator (Prof. Andrew Grant), not involved in the participant recruitment or data analysis.

#### Participant allocation

As participants signed up for the study, they were sequentially assigned a random number from the pre-generated list. A deterministic allocation rule was implemented so that those assigned an even number were allocated to the ESRT group, while those assigned an odd number were allocated to the control group. This method ensured each participant had an equal chance of being placed in either group.

#### Allocation concealment

To prevent selection bias, the allocation process was conducted without informing the researchers or participants of their group assignment in advance. The random sequence was maintained by Prof. Grant, who only provided group assignments after participants were registered in the trial.

### Quantitative analyses

#### Primary analysis

AAQ2, CD-RISC 10, and PSRS summary scores were calculated for each randomised participant at T1, T2, and T3. A priori quantitative analyses included 3 (time) x 2 (group) mixed effects ANOVAs on each outcome measure. Analysis focused on interaction effects to determine the degree of change over time and if any change differed by group. Effect sizes were displayed as partial eta squared values and interpreted according to Cohen’s classification of small (< 0.06), medium (0.06–0.14) and large (> 0.14) effect sizes, consistent with recent ESRT research [32, 67]. Bayes factors were also reported to illustrate the degree of support for findings [65, 68, 69], and were interpreted as follows: values of 1 indicated ‘no evidence’, values of 1–3 ‘anecdotal evidence’, values of 3–10 ‘moderate evidence’, values of 10–30 strong evidence, values of 30–100 very strong evidence, and values exceeding 100 extreme evidence (*BF*_10_). Follow-up pairwise comparisons were performed on significant interaction effects to identify the strength and direction of effects. Bonferroni corrections were applied to mitigate Type 1 error. Cohen’s *d* was used to measure effect sizes with reference to Cohen’s benchmarks of small (*d* = 0.2), medium (*d* = 0.5) and large (*d* = 0.8) effects sizes, consistent with recent ESRT research [33, 67]. An intention-to-treat (ITT) sensitivity analysis was performed on significant findings by carrying last observations forward from missing data entries and rerunning ANOVA models. In the interests of consistency and transparency, we included analyses of the original dataset al.ongside an intention-to-treat analysis, which prior research has not reported [23, 31, 33].

#### Post-hoc analysis

Post-hoc exploratory analyses on resilience and stress reactivity were conducted to determine whether ESRT had a significantly greater impact on participants who displayed low versus medium-high resilience levels at baseline. A median split was performed on T1 resilience scores to produce a third independent variable (baseline resilience) for analysis in a 2 (group) x 3 (time) x 2 (baseline resilience) mixed effects ANOVA. Follow-up pairwise comparisons were performed on significant results.

### Qualitative analysis

Exit evaluation responses were summarised in Excel, while interviews and focus groups were auto transcribed by Zoom, reviewed, and ratified. Transcripts and evaluation responses were anonymised and uploaded to NVivo to undergo a reflexive thematic analysis [70, 71]. This approach recognises the unique experience, skills, and expertise of the researcher, which can be utilised to produce meaningful insights into the qualitative data. Coding was carried out through a critical realist epistemological perspective [72, 73]. After thoroughly engaging with and re-examining the data, we applied a cyclical process of coding participant transcripts to identify noteworthy elements within the dataset. This process led to the development of initial themes which underwent repeated evaluation and refinement in consultation with co-authors to ensure a clear delimitation and distinctiveness of themes. Key quotes were selected to represent each theme and provide insight into participants’ experience. The results and discussion were reviewed by co-authors to craft a narrative to demonstrate the breadth and depth of participant experience and situate the findings within the broader context of the data.

#### Reflexivity and positionality statement

Qualitative data collection and analysis was conducted by a single co-author (GB), an early career researcher and qualified medical doctor with a decade of clinical experience, holding various roles within medical education. GB’s personal journey of recovery from a life-altering incident sparked a deep interest in resilience and positive psychology, particularly in relation to post-traumatic growth. Her clinical background, particularly in high-burnout specialties such as emergency medicine and general practice, deepened her interest in interventions targeting burnout, both among students and medical professionals. GB’s dual perspective as a medical educator and clinician brings a nuanced understanding of the unique stressors faced by medical students.

### Integration of quantitative and qualitative findings

To meet the demands for more robust integration methods within mixed-method research [74, 75], we produced a joint display Table [76] and employed a triangulation protocol [52, 77] to enhance the credibility and validity of our findings by exploring convergence, complementarity, and dissonance between quantitative and qualitative findings. Outcomes from our quantitative analyses were compared with themes from our qualitative analysis to identify possible explanations for and contextualisation of quantitative results.

## Results

### Recruitment & attrition

An attrition rate of 52% was observed between randomisation (*n* = 98) and T3 (*n* = 47). Numbers of third (*n* = 7, 14.9% of final sample) and fourth (*n* = 3, 6.4% of final sample) year students were especially low at T3. Participants with missing data for CD-RISC 10 scores were identified (*n* = 8, 17%), leaving a sample size of 39 for the resilience measure. Anonymous online exit evaluations had a response rate of 75% (*n* = 25). Several ESRT participants agreed to take part in semi structured interviews (*n* = 12), and end of study focus groups (*n* = 11). Demographic information is displayed in Table 2, while a participant flow diagram is provided in Fig. 1.

**Table 2:** Demographic information

Characteristic	Quantitative Analyses			Qualitative Analysis	
	^a^AAQ2 (*n* = 47)	^b^PSRS (*n* = 47)	^c^CD-RISC 10 (*n* = 39)	Interviews (*n* = 12)	Focus Groups (*n* = 11)
Sex (Male, Female)	12, 35	12, 35	10, 29	1, 11	2, 9
Age (*M*,* SD*,* range)*	27.09, 6.37, 21–49	27.09, 6.37, 21–49	27.05, 6.40, 21–49	25.67, 4.88, 21–40	25.33, 5.31, 22–40
Year of Study N (1, 2, 3, 4)	20, 17, 7, 3	20, 17, 7, 3	15, 15, 6, 3	9, 1, 1, 1	10, 1, 0, 0

^a^Acceptance and Action Questionnaire – 2

^b^Perceived Stress Reactivity Scale

^c^Connor-Davidson Resilience Scale: 10 Item Version

### Quantitative findings

Data from AAQ2, CD-RISC 10, and PSRS questionnaires met al.l necessary assumptions for ANOVA. Descriptive statistics are displayed in Table 3. JASP datafiles are provided on the Open Science Framework [59].

**Table 3:** Descriptive statistics

Psychological Flexibility (AAQ2)	Total sample (*N* = 47)	Intervention (*n* = 29)	Active Control (*n* = 18)
T1 (M, SD, range)	24.98, 7.02, 11–42	25.83, 7.37, 13–42	23.61, 6.39, 11–37
T2 (M, SD, range)	21.47, 6.46, 8–34	21.76, 6.39, 9–34	21.00, 6.73, 8–34
T3 (M, SD, range)	20.77, 6.39, 7–39	19.52, 5.27, 9–30	22.78, 7.61, 7–39

Resilience (CD-RISC)	Total sample (*N* = 39)	Intervention (*n* = 23)	Active Control (*n* = 16)
T1 (M, SD, range)	27.82, 5.12, 17–39	28.26, 5.26, 18–39	27.19, 5.02, 17–35
T2 (M, SD, range)	30.54, 5.15, 18–40	31.17, 4.39, 23–40	29.63, 6.12, 18–40
T3 (M, SD, range)	29.08, 5.73, 14–39	30.30, 4.67, 20–39	27.31, 6.76, 14–36

Stress Reactivity (PSRS)	Total sample (*N* = 47)	Intervention (*n* = 29)	Active Control (*n* = 18)
T1 (M, SD, range)	24.94, 6.45, 12–38	26.07, 6.78, 12–38	23.11, 5.57, 13–33
T2 (M, SD, range)	20.43, 7.12, 4–39	21.10, 7.46, 9–39	19.33, 6.61, 4–31
T3 (M, SD, range)	19.79, 6.67, 6–34	20.66, 6.72, 8–34	18.39, 6.52, 6–31

*M* Mean, *SD* Standard Deviation

#### Primary analyses

##### Psychological flexibility (AAQ2)

**Fig. 2:**
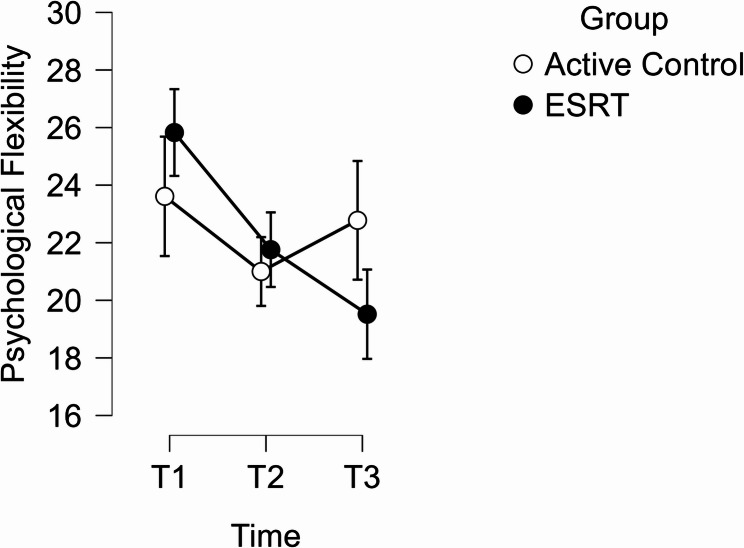
Mean Psychological Flexibility Scores Across Time

ANOVA results revealed a significant interaction between time and group, *F* (2, 90) = 6.30, *p* =.003, *n*^*2*^_p_ = 0.123, *BF*_*10*_ = 18.18. The *BF*_*10*_ value indicated strong evidence in support of this finding. For the ESRT group, psychological flexibility significantly improved by a mean of 4.07 points (15%) between T1 and T2, *t* (28) = 4.11, *p* =.001, *d* = 0.62 (95% CI: 0.12 to 1.11), *BF*_*10*_ = 171.26. Between T2 and T3, scores improved by a further 2.24 points (10%), resulting in a total mean improvement of 6.31 points (24%) between T1 and T3, *t* (28) = 6.38, *p* = < 0.001, *d* = 0.96 (95% CI: 0.41 to 1.51), *BF*_*10*_ = 4969.94. This was nearly one standard deviation from their baseline scores (SD = 7.73). Figure 2 illustrates mean scores across time, by comparison to active controls.

An ITT sensitivity analysis was performed by returning to the original sample of T1 participants (*n* = 73) and carrying the last observations forward. After carrying 16 T1 values and 24 T2 values forward, assumption checks were repeated. T1 data produced a significant Shapiro-Wilk value (*p* =.012), though both distribution and Q-Q plots indicated sufficient normality to continue analysis. The assumption of sphericity was violated and controlled for using Greenhouse-Geisser’s correction.

Congruent with the previous ANOVA, a significant interaction effect between time and group was observed, *F* (1.69, 119.98) = 4.86, *p* =.013, *n*^*2*^_p_ = 0.064, *BF*_*10*_ = 6.12. Follow-up pairwise comparisons indicated a significant improvement in psychological flexibility among the ESRT group between T1 and T2, *t* (46) = 4.01, *p* =.001, *d* = 0.39 (95% CI: 0.08 to 0.69), *BF*_*10*_ = 108.08, and between T1 and T3, *t* (46) = 5.87, *p* = < 0.001, *d* = 0.57 (95% CI: 0.24 to 0.89), *BF*_*10*_ = 1838.00.

##### Resilience (CD-RISC 10)

ANOVA results revealed no significant interaction between time and group, *F* (2, 74) = 0.68, *p* =.51, *n*^*2*^_p_ = 0.018, *BF*_*01*_ = 4.34. The Bayes factor indicated moderate evidence to support this finding.

##### Stress reactivity (PSRS)

ANOVA results revealed no significant interaction between time and group, *F* (2, 90) = 0.24, *p* =.78, *n*^*2*^_p_ = 0.005, *BF*_*01*_ = 6.57. The accompanying Bayes factor indicated moderate evidence in support of this finding.

#### Post-hoc analyses

Median T1 resilience scores (*Mdn* = 28) were used to perform a median split on CD-RISC 10 survey responses. The median score converged with 25th percentile population scores (25th % = 29) [54]. Consequently, baseline resilience scores < 28 were coded as ‘low’ (*n* = 15) while values ≥ 28 were coded as ‘medium-high’ (*n* = 24). Baseline resilience scores were then added as a third independent variable of the ANOVA model.

**Fig. 3:**
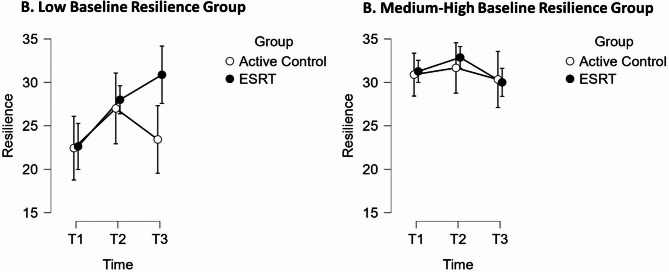
Comparison of Mean Resilience Scores between Baseline Resilience Groups

Results for psychological flexibility indicated no significant three-way interaction between time, group, and baseline resilience, *F* (2, 70) = 3.21, *p* =.057, *η²*_*p*_ =.084. For resilience scores, a significant interaction between time and baseline resilience was observed, *F* (2, 70) = 6.70, *p* =.002, *η²*_*p*_ =.16. Additionally, there was a significant three-way interaction between time, group and baseline resilience, *F* (2, 70) = 4.43, *p* =.015, *η²*_*p*_ =.11. Subsequent *t*-tests revealed that participants with low baseline resilience scores who underwent ESRT exhibited no significant increase in resilience between T1 and T2, *t* (7) = 3.28, *p* =.11, *d* = 1.21 (95% CI: 0.18 to 2.60), *BF*_*01*_ = 0.03. However, a significant increase was observed between T1 and T3, *t* (7) = 5.04, *p* = < 0.001, *d* = 1.86 (95% CI: 0.35 to 3.37), *BF*_*10*_ = 12.72. By contrast, those with similarly low baseline resilience scores in the active control group showed no significant increase in resilience between T1 and T3, *t* (6) = 0.57, *p* = 1.00, *d* = 0.23 (95% CI: −1.16 to 1.61), *BF*_*01*_ = 2.58. See Fig. 3 for a graphical comparison between low and medium-high baseline resilience groups.

**Fig. 4:**
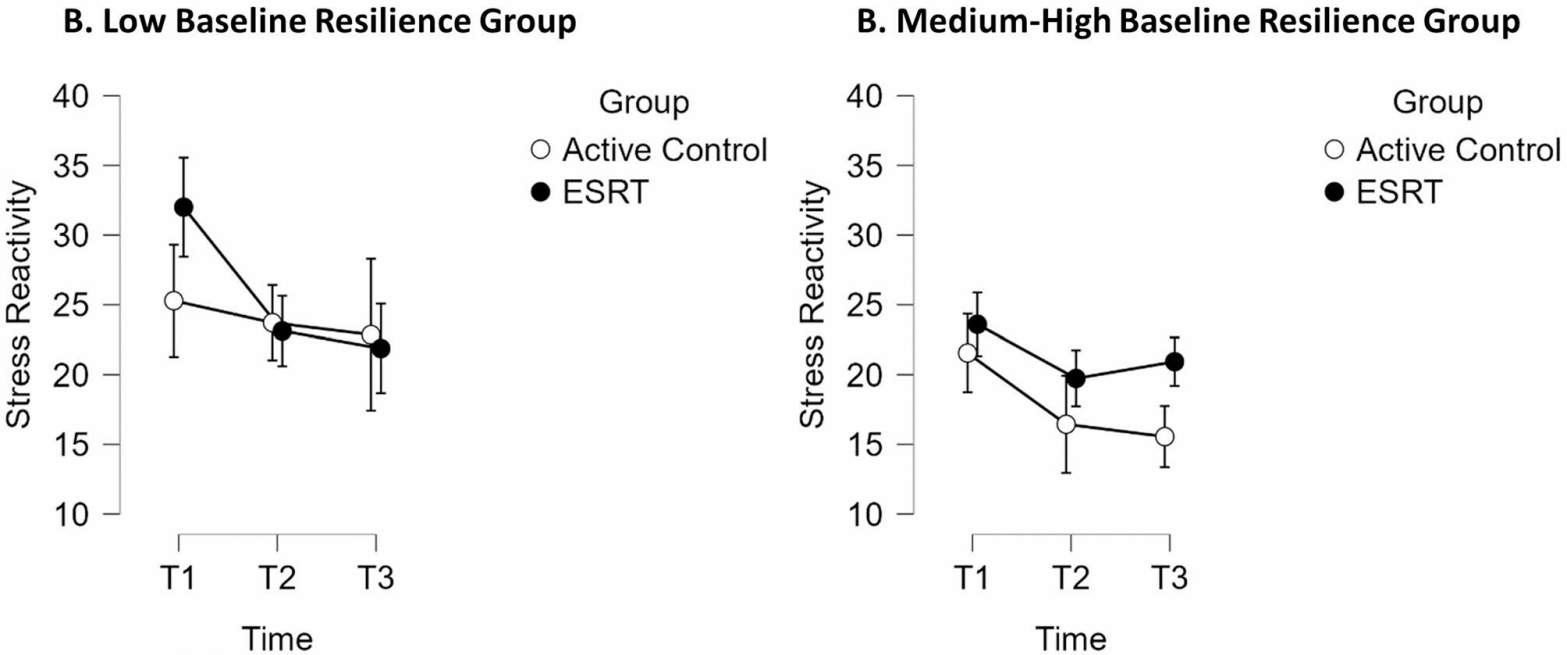
Comparison of Mean Stress Reactivity Scores between Baseline Resilience Groups

Results for stress reactivity revealed a significant three-way interaction effect between time, group and baseline resilience, *F* (2, 70) = 5.06, *p* =.009, *η²*_*p*_ =.126. Subsequent *t*-tests indicated that those with low baseline resilience scores who underwent ESRT displayed significant decreases in stress reactivity between T1 and T2, *t* (7) = −4.61, *p* =.001, *d* = 1.38 (95% CI: 0.17 to 2.60), *BF*_*10*_ = 25.79. This effect was sustained between T1 and T3, *t* (7) = −5.26, *p* = < 0.001, *d* = 1.58 (95% CI: 0.32 to 2.84), *BF*_*10*_ = 20.82. However, those with medium-high baseline resilience who underwent ESRT exhibited a non-significant decrease in stress reactivity between T1 and T2, *t* (14) = −2.75, *p* =.50, *d* = 0.60 (95% CI: −0.22 to 1.43), *BF*_*01*_ = 0.33. A similar result was observed between T1 and T3, *t* (14) = −1.90, *p* = 1.00, *d* = 0.42 (95% CI: −0.39 to 1.22), *BF*_*01*_ = 0.80. See Fig. 4 for a graphical comparison between the low and medium-high baseline resilience groups.

### Qualitative findings: reflexive thematic analysis

No Zoom-related technical difficulties were reported. Six themes emerged through analysis of exit evaluations, interviews, and focus group transcripts. Qualitative datafiles are provided on the Open Science Framework [59]. Themes included:

- The “relentless” pressure of medical training.
- A particularly difficult time during the pandemic.
- From scepticism to confidence in ESRT.
- ESRT enhanced stress management.
- ESRT enhanced known resilience factors.
- A call for integration.

#### The “relentless” pressure of medical training

Students consistently reported distress among themselves and peers, which was attributed to individual and institutional factors, including the personality types of those studying medicine, the high demands of the curriculum, and a stigmatising culture within the medical profession. One student disclosed that her ongoing struggle culminated in “having to” contact her GP and begin antidepressant treatment. Upon reflection she noted, “it was quite clear that it was a mixture of different things. A big thing was my academic stress.” This sentiment was echoed throughout the sample, who admitted to struggling with the transition from undergraduate studies to graduate-level medical education. One student highlighted the pressure associated with the demand for constant knowledge acquisition:

I’m used to knowing lots [on] a subject but in medicine there’s always [such a] vast amount, like you need to know so much. You always feel like you should be looking up something or checking something, like that feeling of not knowing is constant. (Student 3, focus group A1)

Beyond curricular pressures, several students agreed that the medical profession attracts a certain “type” of individual who is prone to competitiveness, self-criticism, and distress. One student noted the cohort’s competitive nature and concurrent maladaptive perfectionism: “Everyone is quite highly strung and quite competitive. There’s … an undercurrent of competition, which is quite subtle, but it can be quite difficult to ignore.” Another student noted, “I don’t think it’s any secret that medics are quite… generally quite proud, quite competitive. I know a lot of people in my year just keep bashing out until they’re completely burnt out.” Accompanying this competitiveness was the sense that they were just “scraping” a pass.

The curricular demands and accompanying stress led to a de-prioritisation of self-care activities and a “pushing onto the background all those things that you enjoy and all those things that are good for your mental health.” One student admitted, “I just wasn’t looking after myself at all.” This de-prioritisation was seemingly perpetuated by feelings of guilt when performing leisure activities, particularly during weekdays. One student asserted that, as adult learners, “we know when to spend our time learning and when to spend our time, you know, revising… and we know we should spend our time on our mental health, or on our physical health.” Nevertheless, students ruminated over how ill-equipped they were to effectively manage their stress under such high demands.

#### A particularly difficult time during the pandemic

Students continually acknowledged record low staffing relating to the COVID-19 crisis as negatively impacting on their placement experience and as a contributing factor to anxiety around their impending career. There were claims that during placement, students were used to support service provision due to a disparity between the numbers of patients and the availability of staff. One student said, “it’s just a bit terrifying when you get to the point of actually being a doctor, because you know that”. Students alluded to the mistreatment of medical students on clinical placement. They reported conflict with senior staff and a fear of requesting time off for mental health concerns:

It’s not necessarily an easy thing to do, because, you know, you don’t know who you’re talking to. You don’t know if the person that you’re delivering that information to is going to be receptive [..] you could just be called unprofessional. (Student 5, focus group A1)

One student claimed that the cultural taboo surrounding burnout and wellbeing was “perpetuated by this bitterness that [qualified doctors] don’t have the tools.” Students also acknowledged the role of the COVID-19 pandemic in exacerbating distress:

I found last year so difficult, with COVID as well, I found I was getting quite upset quite easily and so I thought a bit of extra help might be good … because well you never know when things could crop up again. (Student B, interview)

Additionally, there was agreement among the sample that their curriculum was unable to perceive them as anything other than medical students, failing to allocate sufficient time to extracurricular commitments. Several students reported having to undertake paid work to finance their studies and felt this was not factored into placement allocations. Other students pointed to a generational anxiety relating to an economic downturn and cost-of-living crisis.

#### From scepticism to confidence in ESRT

In the face of this distress, pre-intervention scepticism of ESRT was widely reported. Students reflected on the ineffectiveness of their curriculum’s current ‘Preparation for Clinical Practice’ sessions, designed to manage burnout and prioritise wellbeing:

They’re kind of like, ‘oh, yeah, we need more people to attend. This is gonna’ support you in how you deal with burnout’. But people aren’t attending because it’s not gonna’ be examined and everyone’s burnt out trying to do all the stuff that they’re trying to do at the moment. [..] And you’re like, ‘okay, probably just better to stay at home, I’d be less burnt out’ [..]. (Student 6, focus group A1)

This sentiment echoed throughout the focus group, who expressed cynicism regarding wellbeing interventions. Reflecting further, they pointed to a lack of understanding and negative preconceptions about mindfulness, highlighting a potential source of the recruitment difficulties we faced in our study. Critically, however, students reported dramatic shifts in their appraisals of ESRT before and after the intervention. One student said, “I was sceptical at first, but it really makes a difference.” Another explained, “maybe I was a little bit sceptical before. But now that, like I’ve been through it, I think that I’ve taken more from it than I thought I was going to.” For several students, it was “seeing the benefit of how [they] felt after class” and “knowing that practicing can bring [them] clarity and stress-relief” that encouraged continuing practice.

#### ESRT enhanced stress management

Students reported improvements to their stress management, which centred around a change in their relationship to stress. One student said, “I learnt a lot about myself and how best to deal with stress. I feel more able to deal with stress and be in touch with my emotions.” Some described a “mental frame shift”, while others reported a better “understanding” of stress. This ability to recognise stress allowed them to deal with it “before it becomes overwhelming”. Beyond learning to better identify their stress, students reported an ability to “step away from it” or, “put the stress more in proportion … with a more realistic view.” One student noted a lowered level of stress after just two weeks:

I found it starting to take effect a lot sooner than I thought it would have. Er, so it was a bit hard initially to get into the routine of the meditation, but I think it was about two and a half weeks in and I just felt like my baseline level of stress was a lot lower. (Student B, interview)

Others noted an improved ability to manage their physiological arousal: “I found it helps me to decrease my heart rate a little in stressful situations.” They also noted a decrease in maladaptive tendencies, with one student noting that “[getting] questions wrong in clinics and things doesn’t play on my mind quite as much as it would have.”

#### ESRT enhanced known resilience factors

The majority of students looked forward to ESRT sessions and experienced positive changes in affect and motivation to practice ESRT skills. Positive changes included greater feelings of autonomy and confidence in their situation. One student who struggled with imposter syndrome and a sense of uncertainty over their place in medicine showed a renewed sense of confidence and commitment by the end of the intervention, stating, “I feel more confident in myself, I know what I’m doing, I know why I’m here. [..] I know now what I need to do if things get like that again.”

Students also reported an increased ability to stay present and engage with and manage their feelings, thoughts and emotions (i.e., psychological flexibility). One student explained:

I was able to separate my thoughts from my actions, and was able to appreciate what I was thinking and why I was thinking it, and I wasn’t scared when negative thoughts came, I was able to deal with them [..]. (Student D, interview).

Another reported:

I feel much more able to ground myself in the present and to relax after even stressful days on placement. I feel more able to be fully present in tutorials and placement learning opportunities such as clinics, which helps me to get more out of them and learn more. I also feel more resilient and am able to reflect on and act on criticism in a constructive way, and even when criticised in an unprofessional way I have been able to respond calmly with professionalism and move the conversation forward. (Anonymous exit evaluation)

One student described their increased sense of presence during their clinical skills practice: “I’m just taking it step by step and being more mindful, and not worrying about what’s going to happen next, or what’s happened before.” This helped them to consciously decide on “the weight [they] give to thoughts”. Students’ descriptions hinted at an increased capacity for cognitive reappraisal, perceiving previously stressful circumstances as momentary challenges: “I’ve been far more just accepting of [things], and just, you know, deal with what comes up. And also, I just feel a bit stronger to deal with it, stronger in myself.” Additional improvements were identified in sleep, cognitive functioning, academic performance, general wellbeing, and interpersonal relationships. This included handling loss in the context of a relationship breakdown, and enhancements in active listening, communication, and candour regarding internal emotional states.

#### A call for integration

Despite the effectiveness of ESRT, students overwhelmingly voiced concern relating to their careers. While students appreciated the institutional efforts to raise awareness of wellbeing, the perceived lack of practical action left them feeling frustrated:

[..] they like to talk about self-care, because you see all these posters up in the break rooms and in the toilets and so, hopefully, that’s a start that people are becoming aware of it, but I don’t think we’re actually seeing it yet. (Student 9, focus group B2)

One student called for a better “understanding of the limitations of enhanced resiliency, compared to dealing with [the] problems in the system”. Another argued that improving the wellbeing of students demands more systemic changes:

Probably on an individual level people can appreciate the benefit. But then it’s just practically implementing that, and I think you kind of need the organisational backing to do that really, which isn’t there at the minute from the NHS [National Health Service] and the med school. (Student 7, focus group B2)

One student pointed to the culture of medicine, which they believed to be at odds with prioritising self-care:

I think the whole culture around the NHS is based around you giving yourself up to help patients and that is reflected in the course and in everyone’s attitude generally. And that’s a culture shift that I don’t think is going to change anytime soon. The NHS is built on the goodwill of people at their own detriment and I think that having self-care inside that narrative doesn’t really match up. So I’m not surprised that organisations within medicine aren’t huge on promoting self-care as a priority. (Student 10, focus group B2)

Though ESRT was seen as a viable option, students suspected that recruitment challenges were due to a perceived lack of time. They acknowledged that stress management interventions are “definitely something that should be maintained” but that implementation creates a “burden” forcing them to “give up some of that valuable time in order to do something that they don’t know is necessarily going to actually work for them.” One student explained, “the time to be able to do it is just really not there” because many students have already “dropped [extracurricular activities] because they can’t keep up with extra things alongside their workload”. Consequently, a consensus emerged that ESRT should be integrated into their academic curriculum:

It would be good to actually see integration of this in course time, where the [university] understands that it actually is part of what makes you a better doctor rather than ‘oh, this is cool, but do it in your own time’. (Student 8, focus group B1)

The most prominent feature of student evaluations was a feeling that “if it was integrated into the course, [it] would be a lot more accessible.” Additionally, the abbreviated nature of ESRT was considered appropriate for integration, making it “quite suitable, because it is manageable.” As one student explained, “if people get benefit just from that little bit then I definitely think it’s useful… you know, something obviously needs to be done because people are struggling right now. That goes without saying.” Students generally agreed that this relatively small time-commitment to the development of stress management could be practically implemented into the curriculum, significantly improving their capacity to cope with the demands of their training.

### Integration of quantitative and qualitative findings

To identify the extent to which our quantitative and qualitative findings overlapped, an integrated results matrix was developed (see Table 4). Student responses largely converged with quantitative findings, despite an open-ended focus on student experience in the qualitative research component.

**Table 4:** Dual display table focusing on key findings from quantitative and qualitative analysis

Quantitative Results	Qualitative Results	Exemplar Quote
Psychological Flexibility significantly improved in ESRT users by post-intervention (*p* =.001, *d* = 0.62) and at six month follow-up (*p* = < 0.001, *d* = 0.96).	ESRT users reported an increased ability to stay present and engage with and manage their feelings, thoughts and emotions (i.e., psychological flexibility).	*Interview*,* Student D*: “I was able to separate my thoughts from my actions, and was able to appreciate what I was thinking and why I was thinking it, and I wasn’t scared when negative thoughts came, I was able to deal with them.”
ESRT users with low baseline resilience showed no significant improvements in resilience by post-intervention (*p* =.11, *d* = 1.21). However, by the six months follow-up, significant improvement was observed (*p* = < 0.001, *d* = 1.86).	ESRT users reported an increased ability to adapt to and recover from stressors (i.e., resilience).	*Anonymous exit evaluation*: “I also feel more resilient and am able to reflect upon and act upon criticism in a constructive way, and even when criticised in an unprofessional way I have been able to respond calmly with professionalism and move the conversation forward.”
ESRT users with low baseline resilience showed significant decreases in perceived stress reactivity at post-intervention (*p* =.001, *d* = 1.38) and six months follow-up (*p* = < 0.001, *d* = 1.58).	ESRT users reported improvements in the capacity to regulate physiological and psychological stress responses (i.e., perceived stress reactivity).	*Interview*,* Student B*: “I think it was about two and a half weeks in and I just felt like my baseline level of stress was a lot lower.” *Anonymous exit evaluation*: “I learnt a lot about myself and how best to deal with stress. I feel more able to deal with stress and be in touch with my emotions.”

#### Psychological flexibility

The quantitative results showed a significant increase in psychological flexibility for ESRT participants at post-intervention (*p* =.001, *d* = 0.62) and six months follow-up (*p* = < 0.001, *d* = 0.96). These results converged with findings from our thematic analysis, which suggested that ‘ESRT Enhanced Known Resilience Factors’ and found that students self-reported greater flexibility and autonomy in how they handled challenging situations.

#### Resilience

No overall significant change in resilience was observed immediately post-intervention. However, at six months follow-up, students with low baseline resilience showed substantial improvements (*p* = < 0.001, *d* = 1.86). This partially converged with qualitative reports that ‘ESRT Enhanced Known Resilience Factors’. However, students revealed that they experienced ‘A Particularly Difficult Time During the Pandemic’ and that the resulting staffing shortages and economic anxiety placed them under additional stress. These external factors may have hindered immediate improvements in resilience. However, the delayed improvement by follow-up suggests that ESRT may have supported students in developing resilience in the long-term, perhaps as a consequence of moving ‘From Scepticism to Confidence in ESRT’.

#### Stress reactivity

Quantitative data showed no significant reduction in stress reactivity for the overall group. This presented a discrepancy from our qualitative data, which indicated an overwhelming improvement in students’ ability to manage stress. However, low-resilience students experienced significantly lower stress reactivity at both post-intervention (*p* =.001, *d* = 1.38) and at six months follow-up (*p* = < 0.001, *d* = 1.58), indicating partial convergence with our theme, ‘ESRT Enhanced Stress Management’. While the intervention may not have led to broad reductions in stress reactivity across all participants, those with greater initial vulnerability to stress experienced profound benefits in managing their stress reactivity.

#### Attrition

Pre-intervention scepticism, highlighted in the qualitative theme ‘From Scepticism to Confidence in ESRT’, may have contributed to the high attrition rate (52%). Students reported that previous wellbeing initiatives offered by the university were ineffective. This may have led some participants to disengage early, particularly those who felt that their academic commitments outweighed the potential benefits of ESRT. However, for those who engaged with the intervention, the significant improvements in psychological flexibility likely contributed to their post-intervention confidence in ESRT. Our final theme, ‘A Call for Integration’, revealed that students recognised the potential long-term benefits of embedding ESRT into the medical curriculum to improve accessibility and overcome conflicting academic commitments.

## Discussion

Our findings provide the first evidence of the effectiveness of ESRT in graduate-entry medical students, complimenting findings reported in US- and UK-based surgical trainees [23, 30–32]. These findings provide important new evidence for a viable strategy to counter the growing mental health crisis facing such students [1]. Our findings also reinforce the cross-cultural generalisability of ESRT in a UK sample. ESRT was particularly effective at enhancing psychological flexibility, an effect verified by ITT sensitivity analysis and reiterated in qualitative self-reports, suggesting that the intervention supports students to engage with and manage feelings, thoughts, and emotions that could act as a useful tool for enhancing the quality of patient care [78, 79]. Furthermore, the observed change was sufficient to push baseline psychological flexibility scores (*M* = 25.83) below the critical range of 25–28 points associated with psychological distress, although further research is needed to confirm this [53, 80]. Recent research has identified a negative correlation between psychological flexibility and depression, anxiety, burnout, and post-traumatic stress disorder [81], suggesting that flexibility could play an important mediating role between ESRT and reduced PBS. Post-hoc quantitative analyses also revealed that ESRT was effective at increasing resilience and decreasing stress reactivity in those participants who began the intervention with low baseline resilience levels, providing preliminary evidence for the effectiveness of ESRT in vulnerable student populations. Crucially, those with low baseline resilience displayed improvements in resilience at the six-month follow-up. Qualitative themes highlighted the “relentless” pressure of medical training and a particularly difficult time during the pandemic, which may partially explain this delayed effect. Equally, the shift from scepticism to confidence in ESRT that we observed in our qualitative reports suggests that some participants may not have engaged deeply with the intervention initially, potentially dampening early effects. Yet, as students had more time to integrate the ESRT practices, their resilience levels showed significant improvements.

### Strengths & limitations

The principal limitation of this study lies in the reduced sample size and high attrition. These limitations reflect broader challenges associated with pandemic-era research, including the shift to remote learning, limited access to digital infrastructure, and competing academic demands. Qualitative data indicated that time constraints and curricular overload undermined participation, particularly among later-year students. These barriers are consistent with reports from medical students and trainees facing similar pressures [20, 30, 33]. As a result, outcomes may be skewed towards students who were earlier in their training or inclined to engage in wellbeing initiatives.

Despite these challenges, the study offers several methodological strengths. The mixed-methods design enabled integration of quantitative outcomes with student narratives, allowing for a richer and more nuanced understanding of intervention impact. Improvements in psychological flexibility were supported across both strands of data, while delayed effects on resilience were clarified through participant reflections. Robustness was further enhanced through intention-to-treat analyses using last observation carried forward, post hoc subgroup analyses based on baseline resilience, and the reporting of effect sizes and Bayes factors to indicate the strength of evidence. Several outcomes demonstrated large effects and strong Bayes support, lending credibility to findings despite limited power. A six-month follow-up allowed for assessment of sustained change, and while we employed ANOVA for comparability with prior ESRT research, future studies should consider multilevel models to better account for nested data structures [82]. Finally, qualitative feedback highlighted systemic barriers to engagement, suggesting that integration of ESRT into the core curriculum may enhance uptake and help to normalise wellbeing practices within medical training. Early curricular delivery, particularly in the first two years, may offer a strategic opportunity for embedding such interventions.

## Conclusions

Our study revealed the capacity for ESRT to enhance psychological flexibility in graduate-entry medical students, while also increasing resilience and decreasing stress reactivity in the most vulnerable students. Ultimately, this brief yet powerful intervention holds promise for positively supporting medical students and fulfilling the demand for meaningful curricular change. Our findings reinforce cross-cultural generalisability and generalisability across different stages of medical education, though further applications of ESRT in non-Western medical systems are now needed. To advance implementation efforts, it is imperative that future work is focused on coupling individual skill-building interventions with systemic changes aimed at dismantling the barriers that impede engagement. In doing so, we will be paving the path for a more resilient workforce supported by more humane systems, working in synergy to improve healthcare education and clinical practice.

## Supplementary Information

Supplementary Material 1.

Supplementary Material 2.

## Data Availability

Quantitative and qualitative data and analysis files that support the findings of this study have been deposited in the Open Science Framework and are available at: 10.17605/OSF.IO/F58AR.

## Abbreviations

PBS: Psychological and behavioural problems
MBSR: Mindfulness-Based Stress Reduction
MBI: Mindfulness-Based Intervention
ESRT: Enhanced Stress-Resilience Training
AAQ2: Acceptance and Action Questionnaire 2
CD-RISC 10: Connor-Davidson Resilience Scale:10 item version
PSRS: Perceived Stress Reactivity Scale
PSQ: Pre-sessional questionnaire
ANOVA: Analysis of variance
ITT: Intention-to-treat
BF: Bayes factor
NHS: National Health Service
